# Cancer cells as a new source of induced pluripotent stem cells

**DOI:** 10.1186/s13287-022-03145-y

**Published:** 2022-09-05

**Authors:** Azam Shamsian, Roxana Sahebnasagh, Amir Norouzy, Safin Hassan Hussein, Mohammad Hossein Ghahremani, Zahra Azizi

**Affiliations:** 1 Department of Toxicology and Pharmacology, Faculty of Pharmacy, Tehran University of Medical Sciences, Tehran, Iran; 2 Department of Molecular Medicine, School of Advanced Technologies in Medicine, Tehran University of Medical Sciences, No. 88, Italia St, Keshavarz Blvd., Tehran, Iran; 3 Bioprocess Engineering Department, National Institute of Genetic Engineering and Biotechnology (NIGEB), Tehran, Iran; 4 Department of Medical Laboratory Science, College of Science, University of Raparin, Ranya, Kurdistan Region Iraq

**Keywords:** Induced pluripotent stem cells, Cancer cell reprogramming, Induced pluripotent cancer cells, Cancer-iPSCs

## Abstract

Over the last 2 decades, induced pluripotent stem cells (iPSCs) have had various potential applications in various medical research areas, from personalized medicine to disease treatment. Different cellular resources are accessible for iPSC generation, such as keratinocytes, skin fibroblasts, and blood or urine cells. However, all these sources are somatic cells, and we must make several changes in a somatic cell’s transcriptome and chromatin state to become a pluripotent cell. It has recently been revealed that cancer cells can be a new source of iPSCs production. Cancer cells show similarities with iPSCs in self-renewal capacity, reprogramming potency, and signaling pathways. Although genetic abnormalities and potential tumor formation in cancer cells pose a severe risk, reprogrammed cancer-induced pluripotent stem cells (cancer-iPSCs) indicate that pluripotency can transiently overcome the cancer phenotype. This review discusses whether cancer cells can be a preferable source to generate iPSCs.

## Introduction

Remarkable developments in molecular cell biology revealed that the identity of the cells could be partially or entirely altered through specific methods, even after complete differentiation [1]. The ability of cells to dynamically and reversibly change in phenotype and cell identity is defined as cellular plasticity. This process can be triggered in response to various extrinsic and intrinsic specific signals and cues leading to acquiring new features or/and performing new functions. Cellular plasticity has been extensively observed during wound healing, normal development, cancer progression, and metastasis [2, 3]. The plasticity of pluripotency is the ability of stem cells to differentiate into three germ layers, including the endoderm, mesoderm, and ectoderm. Pluripotent stem cells express specific pluripotency markers and form embryoid bodies in vitro and teratomas in vivo [3, 4].

The discovery of induced pluripotent stem cells (iPSCs) [1] has revolutionized the field of stem cell research and provided numerous iPSCs applications from basic research to regenerative medicine. So far, several mice and adult human somatic cells such as neural cells, dental pulps, adipose cells, amnion-derived cells, and pancreatic β-cells have been transformed into iPSCs. These alterations can be achieved using OSKM factors or other combinations offered by non-viral [5] and viral methods [6], such as lentiviral, retroviral, episomal, or Sendai virus [7–9]. OSKM includes four factors-octamer 3/4 (Oct3/4), SRY-box-containing gene 2 (Sox2), Kruppel-like factor 4 (Klf-4), and c-myelocytomatosis (c-Myc) oncogene that is used to induce pluripotency in vitro [1]. Several methods have been developed for iPSC generation by delivering reprogramming factors and utilizing chemical-induced techniques [6]. However, reprogramming mature adult somatic cells into iPSCs is a challenge. During de-differentiation, many specific functional-associated genes should be silenced, and many pluripotency-associated genes should be activated. In the last decade, using small molecules instead of viral vectors increased the efficiency of non-viral somatic-iPSCs, which enhanced their safety for clinics. However, most of these studies have been done on mice [5].

Along with the improvement and advancement of iPSC technology, cancer research fields have also changed. Several interesting questions have been raised regarding reprogramming human cancer cells into iPSCs [10–16]. Due to increasing attention to cancer research and continuous attempts to discover novel treatments, the present review has focused on cancer cells as a source of iPSC generation to improve the efficiency of this approach and introduce a closer starting point to the aim of pluripotency.

## Cancer-iPSCs and other PSCs: a comparison study

Embryonic stem cells (ESCs) are an example of pluripotent stem cells (PSCs) exhibiting the capacity for high pluripotency, clonal expansion, and self-renewal. The first human embryonic stem cell was derived from blastocysts' inner cell mass. After that, several studies have been addressed to investigate the mechanisms of stem cell pluripotency and the differentiation/dedifferentiation status [17, 18]. However, utilizing the ESC is associated with various limitations, including teratoma formation by embryos, immune rejection of allogeneic recipients, restricted number of available donated sources, and ethical problems of blastocyst destruction. These issues decrease the value of ECs in clinical applications[17–19].

Somatic-iPSCs are another example of PSCs that Takahashi and Yamanaka developed in 2006 through different reprogramming methods [1]. The reprogrammed stem cells were named iPSCs which are considered equivalent to ECS due to the exhibition of ESC-like properties such as expression of ECS marker genes, pluripotency, morphology, and proliferation [1, 20]. Pluripotency, self-renewal, immortality, and teratoma formation are inseparable concepts of all sources of iPSCs [12, 21]. Cancer-iPSCs are another source of iPSCs comparable to other PSCs, including ESCs and somatic-iPSCs. Here, we looked for the differences and resemblances among these PSCs (Table1).

**Table 1:** The comparison of characteristics among embryonic stem cells, somatic-iPSCs, cancer-iPSCs, and cancer cells

Characteristics	PSCS			
	Embryonic stem cells	Somatic-iPSCs	Cancer-iPSCs	Cancer cells
Pluripotency	+	+	+	−
Proliferation	+	+	+	+
Self-renewal	+	+	+	−
Immortality	+	+	+	+
Expression of ECS marker genes	+	+	+	−
Availability	−	+	±	±
Ethical concerns related to isolation	+	−	−	−
Telomere elongation	+	+	+	+
Teratoma formation	+	+	+	−
Tumor formation	+	+	+	+

### Pluripotency, self-renewal, and immortality

The differences between iPSCs and somatic cells are controlled by epigenetic processes. The epigenetic status in iPSCs and ECSs includes DNA methylation and changes in histone modifications necessary for the preservation of pluripotency and telomerase activation [6]. Bernhardt et al. showed more similarity between cancer-iPSC and somatic-iPSCs compared to the parenteral cell lines through gene clustering. Their shape was like ESCs and was similar to somatic-iPSCs in the epigenetic profile of methylation sites and epigenetic modifier genes [22]. Telomere elongation is associated with self-renewal and unbridled cancer cell proliferation. Pluripotency in iPSCs could be due to the high telomerase activity [6, 23, 24] or even telomerase-independent telomere elongation mechanisms [6, 24]. Unlike normal somatic cells, cancer cells, cancer-iPSCs, somatic-iPSCs, and ESCs constantly need telomeres elongation to maintain self-renewal capacity [23].

**Fig. 1:**
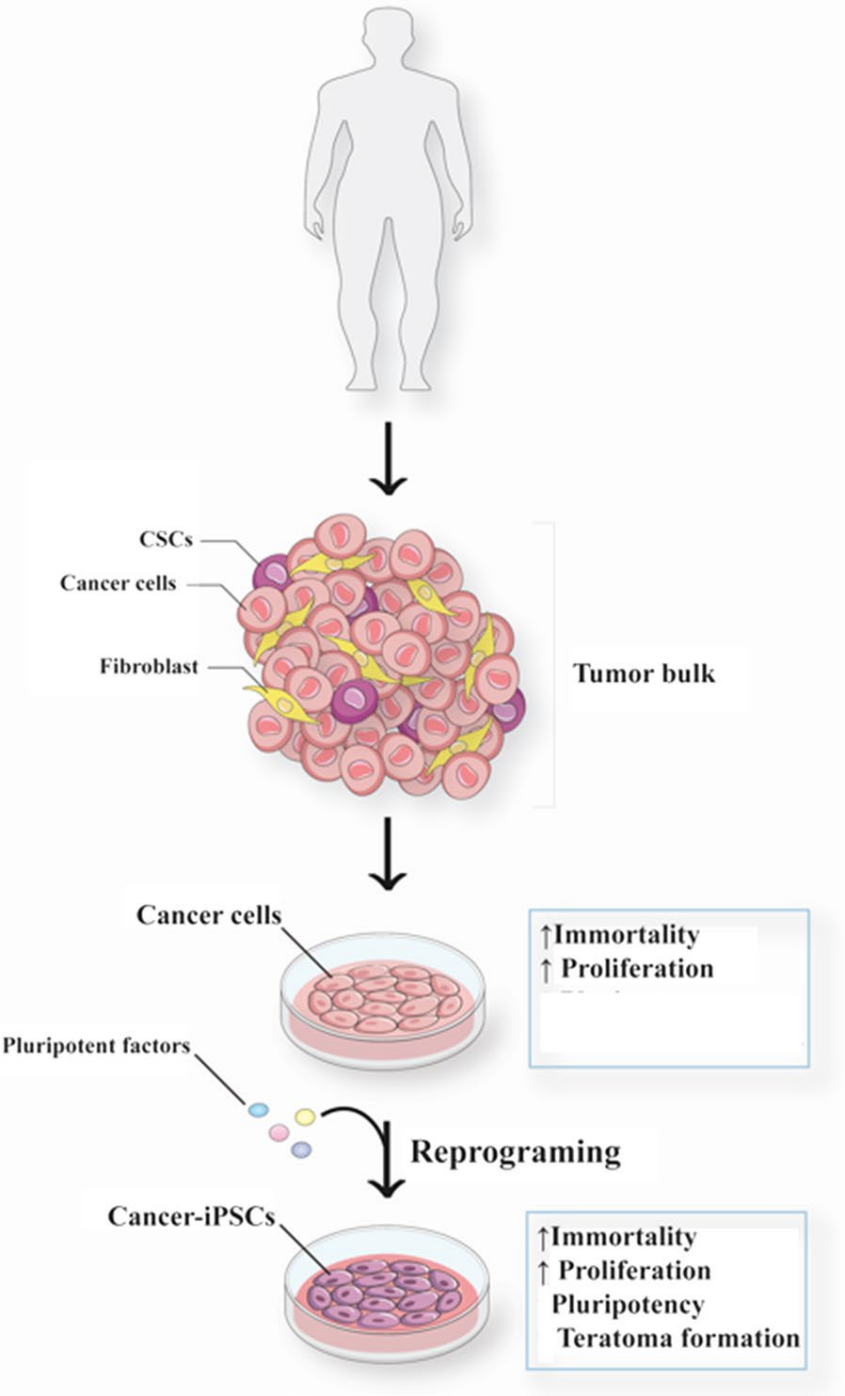
Generation of Cancer-iPSCs: Cancer cells can be isolated from tumor bulk and differentiate into cancer-iPSCs by reprogramming pluripotent factors in vitro. These cells have immortality and proliferation capacity, and they can make tumors. They acquire pluripotency and teratoma formation character during reprogramming stages. Immortality, proliferation, pluripotency, and teratoma formation are four characteristics that can also be seen in CSCs

Like ESCs and somatic-iPSCs, Utikal et al. reprogrammed human and mouse melanocyte cell lines using OSKM factors. Melanocyte-iPSC lines showed pluripotency markers and the ability to differentiate into embryoid bodies in vitro and teratoma formation after injection into the dorsal flank of mice in vivo [12]. There are some reports of cancer-iPSCs teratoma formation [13–15, 25], pluripotency character [9, 14, 26, 27], and differentiation capacity [26] (Fig. 1). Limited reprogramming of cancer cells displays the lower expression of pluripotency genes and increases the probability of cancer reversion [28]. Even fully reprogrammed cancer-iPSCs can reverse into originated cancer cell features over time [29, 30].

Cellular reprogramming involves resetting the epigenetic memories by changing methylation patterns, and establishing a new epigenetic signature leads to a switch chromatin state. A residual epigenetic memory of the donor cell may be maintained in the reprogrammed cells [5]. The accumulation of aberrant DNA methylation and hypermethylation of different gene sets in cytosine–guanine base (CpG) islands occurs during the carcinogenesis stage, affecting iPSCs phenotype [5, 31]. All essential features for pluripotency in reprogrammed cancer-iPSCs, including iPSC-gene expression pattern, morphology, proliferation capacity, and teratoma formation, have not been reported yet [27, 32–35]. Indeed, induction of pluripotency in benign cancer cells is more accessible than in malignant cancer cells [36]. Cancer cell heterogeneity affects reprogramming efficiency and establishing an optimum experimental protocol. Surprisingly, even cancer-iPSCs cannot be obtained from cancer cells completely [11, 12, 28]. Some of these barrier genes are *PRMT6, MXD1,* and *EZH* [28]. The inhibition of these genes might result in obtaining pluripotency in cancer cells. Another study has reported significant epigenetic remodeling in tumor suppressors and oncogenes along with reduced tumorigenicity [27]. Therefore, exogenous reprogramming can erase cancer-related epigenetic abnormalities in cancer-iPSCs. Epigenetic memories differ among various cancer cell types, and the production of iPSCs from cancer cells appears to be cell type-specific. For example, Miyoshi et al. conducted a series of reprogramming studies on twenty gastrointestinal cancer cell lines with eight different results [33]. Studies showed that another problem is incomplete reprogramming significantly reduces the efficiency of iPSC production and generates cells with variable epigenomes and functions. Decreased rate of proliferation, defective differentiation, improper maintenance of pluripotency, and variable DNA methylation and transcription patterns are exhibited by partially programmed cells [37–39]. Overall, full-reprogramming of cancer cells has remained a challenge.

### Teratoma formation and tumorigenicity

The formation of teratoma is one of the methods to investigate iPSC full-reprogramming. iPSCs and ESCs mostly make teratomas. Cancer cells make tumors that can be benign, premalignant, or malignant. Teratoma contains different immature or mature differentiated cells from all endoderm, mesoderm, and ectoderm layers, such as keratinocytes, myocytes, and osteocytes. Most teratomas are not cancerous and are usually formed because of abnormal cell growth in the body. Malignant tumors are cancerous [40]. There are some reports of teratoma formation for cancer-iPSCs derived from Acute Myelogenous Leukemia Cells [13], breast cancer cells [14], prostate cancer cells [15], and osteosarcoma cells [25]. iPSCs can make teratomas quicker and more efficiently than ESCs in vivo, indicating their higher potency in making tumors [41]. Cancer-iPSCs can cause teratomas [12, 13, 15] or tumors similar to their cancer cell origins [42, 43], which might be due to partial reprogramming [43–45]. A study mentioned that partially reprogramming cancer cells could lead to more invasive cancer cells and colonization [36]. Other reports have shown that pluripotency reprogramming hampers tumorigenicity in cancer cells, though it cannot eliminate tumor formation [27, 46].

The safety of any sources of iPSCs in regenerative medicine or therapeutic applications is still controversial. Lack of tumorigenicity and DNA integrity preservation is necessary for using iPSCs in clinics. Kamarda et al. showed that 84% of iPSCs transplanted in mice became weaker or died because of tumor development [40]. Cancer-iPSCs have more potential risk, so their use in therapy should be more cautiously approached. Stimulation or inhibition of specific factors increases the tumorigenicity risk of iPSCs. OSKM factors could provide a condition to promote tumor development on their own because these factors are overexpressed in different cancer cells in a parallel manner [32]. This is especially true for c-Myc [4, 47] and Klf-4 [4]. A method was adopted by Alvarez-Palomo et al. that used synthetic mRNA of some Yamanaka factors along with Cyclin D1 transfection to repair DNA lesions during reprogramming. As a result of Cyclin D1 mRNA function, safer iPSCs are produced, which are sufficiently genetically stable [48]. Upregulation of cyclin-dependent kinase inhibitor 2B or P15 is associated with minimized malignancy during reprogramming dermal fibroblasts into multipotent cells. In contrast, its sustained inhibition leads to tumorigenic, pluripotent, teratoma-forming cells [49]. Mashima et al. expressed herpes simplex virus-thymidine kinase (HSV-TK)/ganciclovir (GCV) and inducible caspase-9 (iCasp9)/AP1903 and made granulocyte–macrophage colony-stimulating factor (GM-CSF)-producing proliferating myeloid cells as an immortal source of antigen-presenting. They used these cells in cancer immunotherapy. Therefore, cell reprogramming in cancer cells equipped with suicide genes as a safety switch introduced a less dangerous cell source of cancer cells for transplantation [50].

Feeder cells can cause tumorigenicity as well. Feeder cells are needed for iPSC culture in vitro and transplanted with iPSCs in vivo. However, it seems that treatment with mitomycin C ceases their proliferation and decreases iPSCs’ ability to form malignancies. The unknown interactions between iPSCs and feeder cells are responsible for converting feeder cells to cancerous type, given that mitomycin C-treated feeder cells are not tumorigenic [40].

Each cell, primary or cell-line cell, has a unique timetable to go through stages of differentiation. Therefore, there is always a mixture of fully differentiated iPSCs, semi-differentiated iPSCs, and undifferentiated cells. Semi-differentiated iPSCs are unstable, and they may choose a different fate instead of our ideal designated one. Transplantation of semi-differentiated iPSCs and undifferentiated cells with fully differentiated iPSCs is unsafe. One way to improve the quality of the transplanted cell population is to irradiate cells with X-rays to undifferentiated iPSCs elimination [51]. In addition, removing the tumor-initiating cells with molecular level-based assays and agents like Notch signaling inhibitors or DNA-methylation analysis qualifies the cell population before clinical application. Detection of undesirable tumorigenic iPSCs could even continue after transplantation using positron emission tomography imaging [52]. Residual risk of malignancy has been remarkably declined by introducing a suicide gene screening system to transplanted iPSC-derived neural stem cells. Expression of Herpes simplex virus type 1 thymidine kinase with ganciclovir administration induces apoptosis in tumorigenic cells without adverse effects in other well-functioning transplanted cells [53].

The elimination of cancer cells by chemotherapeutic agents as a conventional method is undoubtedly an aggressive approach that compels cells to save the identity/existence of the tumor via drug resistance, epigenetic modifications, and reprogramming. During past years, many reports have addressed the role of cell trans-differentiation as another attractive mechanism in tumor progression. This mechanism is a lineage conversion or direct cell reprogramming process that uses lineage-specific transcription factors to change a differentiated cell type into another cell fate and avoid forming a pluripotent state. An alternative method of transdifferentiation combines directed differentiation and partial reprogramming [41, 54]. In this regard, instead of a tumor driving process, trans-differentiation can be considered a tool to encounter cancer cells more gently and convert them to a more benign or at least more sensitive and less aggressive type. Nevertheless, reprogramming itself causes another severe level of stress for cells. The pluripotency in reprogrammed cancer cells might encourage us to pursue this objective, but poor efficiency is less likely to achieve this goal.

Rapino et al. [55] confirmed that transcription factor *C/EBPα* gene expression mediated by an inducer treatment resulted in fading B cell phenotype in most B-cell lines in a wide range of lymphoma and leukemia cell lines. Nevertheless, a macrophage phenotype needed different levels of transgenes to be expressed. Moreover, this study found that one of the high-responsive clones of the Burkitt lymphoma cell line trans-differentiated efficiently to specialized phagocytic macrophages after only 4 days of incubation with 17estradiol-induced C/EBPa. Thus, macrophage trans-differentiation in these cell lines depends on the incubation dose and time. Most importantly, reduced tumorigenicity was an important finding of this study that was achieved through inducer treatment before and after cell injection in vivo. Meanwhile, amongst enforced trans-differentiation observations, albumin-associated lipids have generated adipocyte-like cells in melanoma and MCF-7 breast cancer cell lines. Reduced pigmentation has been considered a suppressed cancer phenotype marker [56].

Cancer stem cell (CSC) features are close to cancer-iPSCs characteristics. Morusin, a cytotoxic agent, also exhibits adipogenesis and apoptosis properties and disturbs tumor formation in glioblastoma stem cells in vitro and in vivo [57]. PPARγ, a ligand-activated nuclear receptor, is significantly expressed in both adipocytes and various cancer cells. However, PPARγ agonists like mycophenolic acid can act as an adipogenic inducer in breast cancer cells [58]. A summary of this section is shown in Table1.

## Factors and pathways in cancer cell reprogramming

Some reprogramming modulators include tumor suppressor protein, cell signaling modifiers, and hypoxia [59–64]. Cellular reprogramming efficiency can be increased by transient inhibition of the phosphatase and tensin homolog protein (*PTEN*) or tumor protein 53 (*TP53*) tumor suppressor gene. The transient inhibition of tumor suppressors increases cell proliferation and inhibits senescence, apoptosis, and cell cycle arrest [59–62]. Miyoshi et al. reprogrammed four different gastrointestinal cancer cell lines to generate the iPSC-like cells by ectopic expression of *KRAS* and *BCL2* oncogenes with the Yamanaka factors and inhibition of the tumor suppressor genes, such as *FHIT*, *TP53*, *PTEN*, *p16Ink4a* by short hair RNAs (shRNAs). These gastrointestinal cancer cell-derived iPSCs showed reduced tumorigenicity [33].

**Fig. 2:**
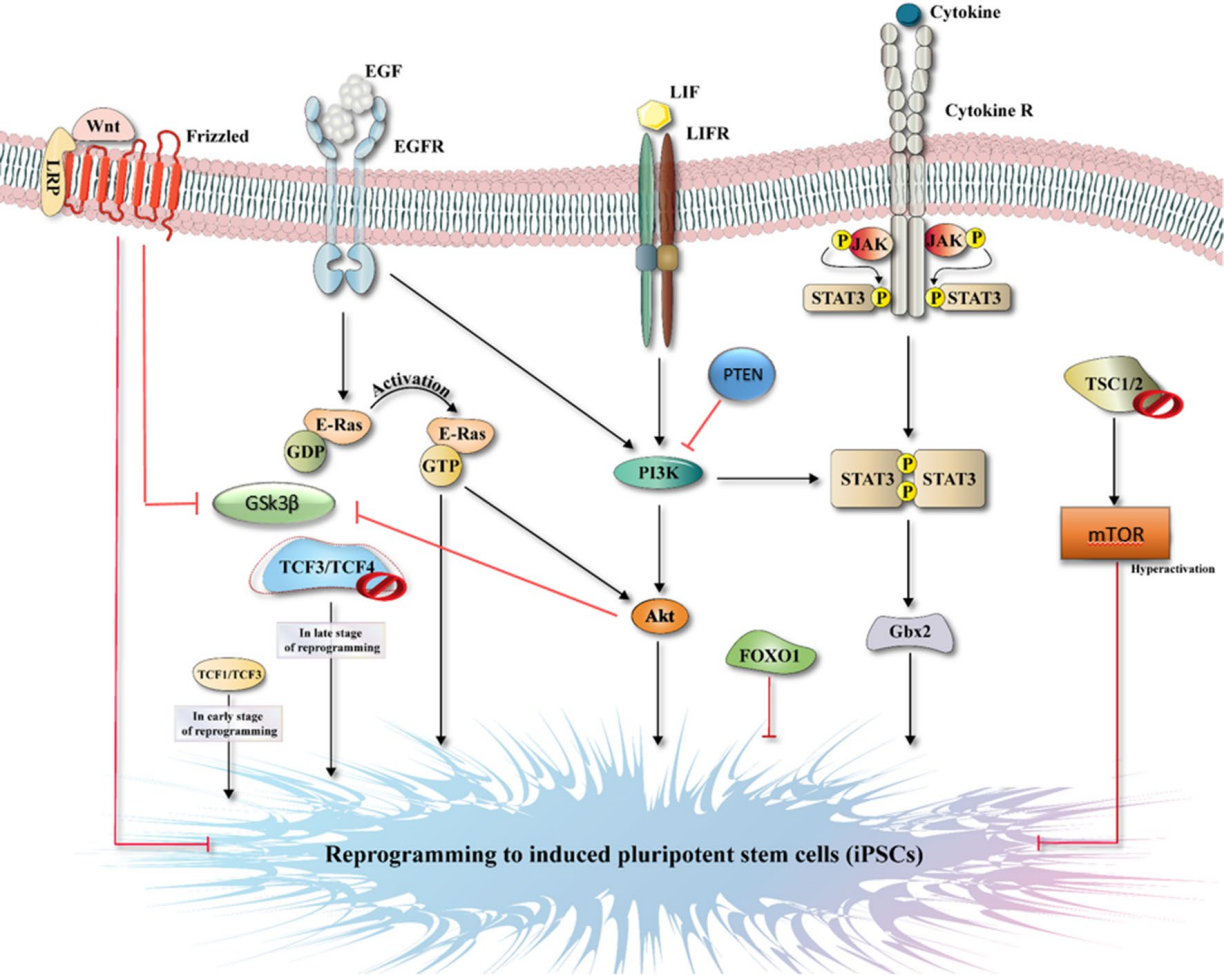
The mechanism of iPSCs generation in vitro. The induction of TCF1/TCF3 is necessary to initiate reprogramming. Then E-Ras, PI3/Akt, and STAT3/Gbx2 pathways activate, and FOXO1 and mTOR pathways are inhibited. In the late stage, Wnt activation starts via deletion of TCF3/TCF4, resulting in iPSCs generation

Reprogramming alters the activation of the signal transduction pathway in parallel with the overexpression of pluripotency factors to prepare the self-renewal conditions. Some well-known signaling pathways involved in self-renewal maintenance and cell survival are deregulated in different cancers, which are supposed to be induced during cell reprogramming [65]. Stemness maintenance of pluripotent stem cells depends on tight regulation of extrinsic signaling pathways, such as GSK3, PI3K/AKT, TGF-β, STAT3/LIF, and FGF/ERK pathways (Fig. 2) [66].

### Wnt/β-catenin pathway

The WNT/β-catenin signaling pathway promotes the reprogramming of cells to iPSCs. Protein-serine O-palmitoyltransferase (PORCN) is an essential processing enzyme that regulates Wnt signaling pathway. Therefore, fibroblasts derived from individuals carrying *PORCN* gene mutations fail cell reprogramming. Ross et al. demonstrated that overexpressing PORCN and WNT signaling are required for reprogramming the fibroblasts to iPSCs. They stated that cell reprogramming depends on the WNT signaling pathway [67]. Activation of the canonical Wnt/β-Catenin signaling pathway is necessary to trigger somatic cell reprogramming and maintain the stemness state. However, this necessity is more evident in the early stages than in the late ones [67, 68]. In the contrary, there is evidence of opposite results, indicating that inhibition of Wnt signaling by TCF1/TCF3 activity in the early stages [69] and Wnt activation via deletion of TCF3/TCF4 in the late stages [70] are requisites for reprogramming. The effect of the Wnt signaling pathway depends markedly on the extent and timeframe of activation during reprogramming. The β-Catenin function supports breast cancer cells to maintain the stem-like subpopulation by reprogramming more differentiated cells [71]. Wnt/β-Catenin and Notch pathways, which indicate a poor prognosis in glioblastoma, are also activated during their primary cells' dedifferentiation and CD133 expression [72].

### MAPK/ERK pathway

The induction of E-Ras expression in somatic cells and OSKM factors enhances reprogramming efficiency, shortens the emergence of pluripotent stem cell colonies, and conserves pluripotency properties after several passages. The level of Akt phosphorylation is transiently increased under the influence of E-Ras during the reprogramming process. FoxO1 activation inhibits iPSC generation, whereas its inactivation as one of the downstream Akt targets does not markedly improve the reprogramming process [73].

### JAK/STAT pathway

It has been shown that increased activity of Akt1 promotes reprogramming via enhancing the activity of Stat3 in coordination with LIF [74]. In this regard, Akt, LIF, and other factors like granulocyte-colony stimulating factor-stimulated GY118F receptor lead to STAT3 activation targets as one of the critical molecular events in reprogramming. Thus, Akt inhibition alone does not prohibit iPSC colonies entirely [75, 76]. Gbx2, as a transcription factor and one of the downstream targets of STAT3, is the final factor in the JAK/STAT3 axis. Gbx2 provides self-renewal in ESC even in the absence of STAT3 and is adequate to redirect epiblast-derived stem cells to reprogram completely [77]. JAK/STAT3 activation is required to attain pluripotency in the late stages [75]. However, a study found that a pulse of temporary early activation yields a similar result to continuous activation [76].

### mTOR pathway

Efficient reprogramming requires strict regulation of signaling pathways' functions, such as the mammalian target of rapamycin (mTOR). The mTOR pathway is affected by only Sox-2 among four OSKM factors to modulate energy metabolism and autophagy for initiation of reprogramming. Either augmented mTOR activity by TSC1/TSC2 knockdown or complete mTOR blockade impairs iPSC generation [78]. However, the reported procedure in the mTOR pathway to accomplish the reprogramming process is the early reduction in the mTOR expression by Sox-2, followed by recovering the mTOR activity to normal levels in the late stages [79]. mTOR inhibition is frequently achieved by defined concentrations of rapamycin, although the exact concentration to receive a desirable response is contradictory [78]. Surprisingly, it is clarified that mTOR upregulation and reduced upstream AMP-activated protein kinase levels are indispensable to converting cancer cells to cancer stem-like cells mediated by Sox-2 overexpression in reprogrammed breast cancer cell lines. Cells are severely sensitive to mTOR levels to initiate and finalize reprogramming.

### Cell cycle phases

Cancer cells tend to accumulate in the proliferative phases, including the S phase [80], due to defective processes of cell cycle checkpoints. In return, cancer-iPSCs are more quiescence [80], and as expected, they shift to the S/G2/M phases again through differentiation [72].

The reprogramming factors are ineffective in regulating the degree of stemness in response to microenvironment changes, even in cell cycle phases. It has been revealed that the inverse correlation of Nanog and Klf-4 in AH130 hepatoma cells regulates the c-Myc levels. Interestingly, these regular changes occur in response to cell cycle activation and O_2_ pressure. It is worth noting that hypoxia increases the Nanog/Klf-4 balance and reduces it in a senescence state and anaerobic condition. So far, none of these factors are detectable in tumor cells and differentiation into some cell lineages [32].

## Applications of cancer-iPSCs

Like iPSCs, a non-cancer cell can acquire pluripotent properties during reprogramming to become a cancer cell. This gives iPSCs a unique status as appropriate cancer models [28, 35, 81]. Exogenous expression of the reprogramming factors changes the epigenome and transcriptome of cells leading to activation of some pluripotency and reprogramming of endogenous regulators [1, 77, 82]. Reprogramming cancer cells into iPSCs is more accessible than de-differentiating mature adult somatic cells into iPSCs due to multiple similarities between iPSCs and cancer cells [83]. In a cancer cell, mutated epigenetic regulators are reprogrammed into oncogenic properties through cancer progression in the body. These epigenetic changes lead to the acquisition of uncontrolled growth and self-renewal properties [83]. However, a somatic cell should exogenously undergo the initiation, maturation, and stabilization process to acquire uncontrolled growth and self-renewal properties outside the body [5, 84]. Numerous studies showed that immortalized cancer cells could be reprogrammed into iPSCs using various techniques, the same as normal somatic cells [10–16, 35, 85].

The similarities between cancer-iPSCs and CSCs bring up an opportunity to discover the intrinsic processes of early stage human cancer cells [11]. They are reprogramming cancer cells back into their original cellular state by the cancer-iPSCs method. This state is an earlier stage of malignant tumor cells that expresses CSC character. So, this gives us a powerful tool for studying the dynamic events in the course of cancer progression mechanisms and understanding the niche that cancer cells are developed.

Cancer-iPSCS can be used as cancer disease models to investigate their biological properties and provide an instrument to do a more comprehensive study. They can generate less tumorigenic cells or chemotherapeutic sensitive ones and determine cancer-related genes, such as oncogenes and anti-oncogenes. Cancer-iPSCs have biomedical applications as well. These cells are individual-specific, which makes them a good candidate for therapeutic drug screening, toxicological studies, drug resistance, and relapse, as well as the identification of early biomarkers and new therapeutic agents.

The investigation of cell–cell interaction between mesenchymal stromal cells (MSCs) and CSCs is achievable using cancer-iPSCs and MSCs. MSCs support tumor progression by constructing an optimum microenvironment for CSCs. These cells have exosome-mediated crosstalk. MSCs can fuse with any cell type, and fused cells could express originated cell type patterns [86, 87]. The fusion of CSCs with MSCs is very controversial because studies reported that they could promote malignant properties [88] or, in contrast, suppress tumorigenicity [89]. Due to the limited number of CSCs in a tumor, generated cancer-iPSCs provide an ideal tool to study MSCs and CSCs interactions.

On the other hand, we have limitations such as different cellular states [28] and heterogeneity of initial populations, which are challenges for generating cancer-iPSCs [90]. In addition, the active non-removable memory of cancer cells is a two-edged sword. It can be a limitation to generate cancer-iPSCs or create an opportunity to research the influence of epigenetic changes on the disease (cancer) on patient by patient (personalized medicine).

**Fig. 3:**
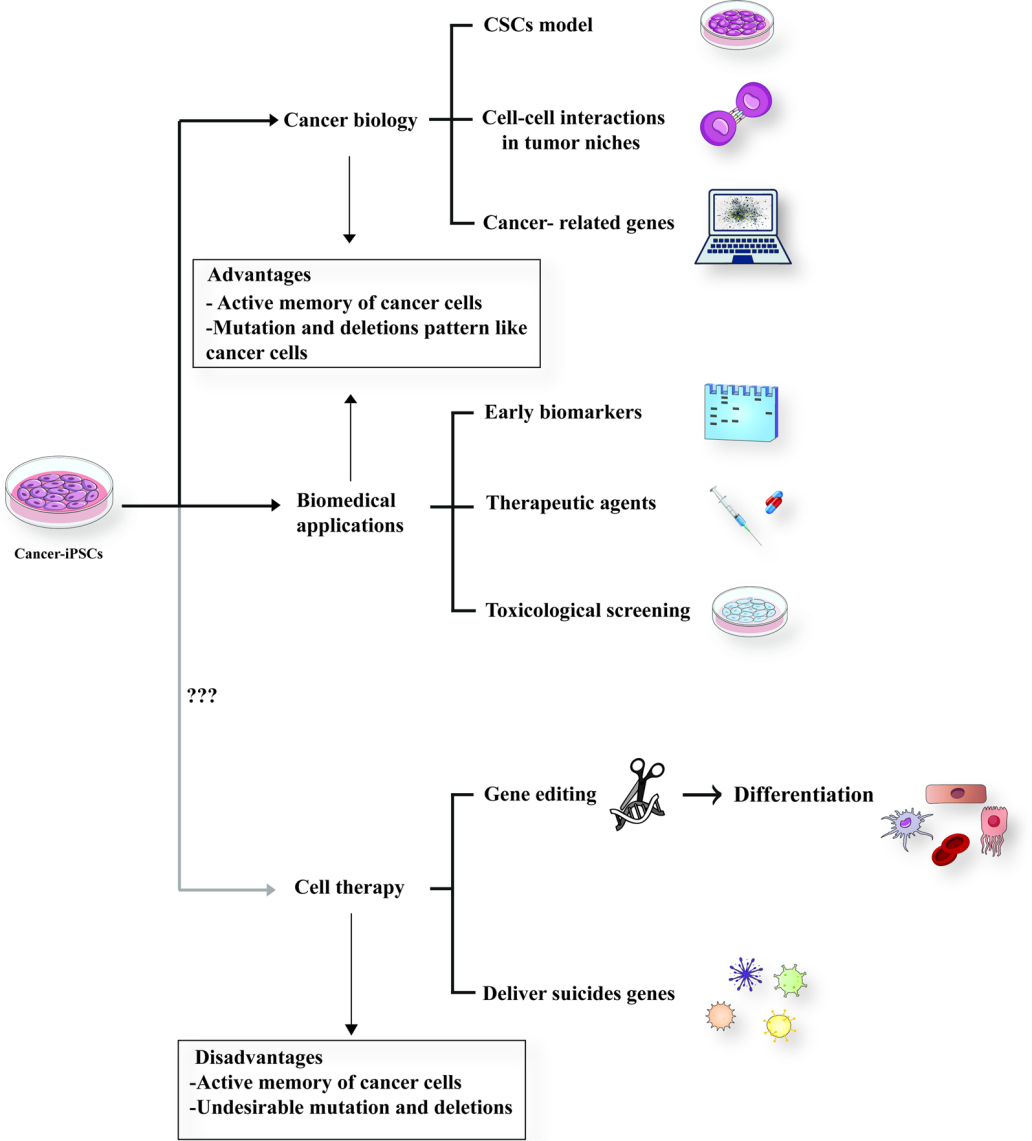
Applications of cancer-iPSCs.Cancer-iPSCs can help study cancer biology by investigating cancer-related genes and cell-to-cell interactions in the tumor microenvironment and are being used as a CSCs model due to the low deficient number of these cells in tumor bulk. They have biomedical applications such as finding new early biomarkers, screening drugs, and introducing new therapeutic agents. These cells can be a candidate in regenerative medicine in suicide gene therapy or some therapy applications after gene editing. However, they are very far from being used in clinics

Cancer-iPSCs have to be used much more carefully in therapy applications. Undesirable mutations and deletions can occur and accumulate during tumor formation. Partial cancer-iPSCs reprogramming [28] or de-differentiation of cancer-iPSCs into originated cancer cells or resistant cells are major obstacles [29, 30, 91]. A summary of this section is shown in Fig. 3.

## Conclusion

Human cancer cell reprogramming into Cancer-iPSCs as a novel strategy has several targets. Cancer-iPSCs can provide a non-limited CSCs pool, generate cancer disease models and help study cancer biology to investigate the development of cancers from origin tissues and cell types. They also have biomedical applications to find early biomarkers and new therapeutic agents, learn about cell–cell interaction in cancer, and answer controversial questions in this field, such as the effect of MSCs on cancer progression and therapy applications.

Cancer cells are resistant to reprogramming, and the mechanisms of this resistance are still unknown. They have biological barriers, including genetic mutations, epigenetic modifications, DNA damage accumulation, and the expression of cancer-related genes, which are the disadvantages of using cancer-iPSCs in regenerative medicine. These restrictions can cause partial reprogramming to generate cancer-iPSCs as well. These limitations can consider the advantages of using cancer-iPSCs in other aspects, excluding regenerative medicine.

## Data Availability

We did not generate any data in this study. Not applicable.

## Abbreviations

Cancer-iPSCs: Cancer-induced pluripotent stem cells
CpG: Cytosine–guanine base
CSC: Cancer stem cell
c-Myc: c-Myelocytomatosis
ESCs: Embryonic stem cells
GCV: Ganciclovir
GM-CSF: Granulocyte–macrophage colony-stimulating factor
HSV-TK: Herpes simplex virus-thymidine kinase
iCasp9: Inducible caspase-9
iPSCs: Induced pluripotent stem cells
Klf-4: Kruppel-like factor 4
mTOR: Mammalian target of rapamycin
MSCs: Mesenchymal stromal cells
Oct3/4: Octamer 3/4
PORCN: Protein-serine O-palmitoyltransferase
PSCs: Pluripotent stem cells
PTEN: Phosphatase and tensin homolog protein
Sox2: SRY-box-containing gene 2
shRNAs: Short-hair RNAs
TP53lf-4: Tumor protein 53
